# Efficacy of simultaneous hexavalent chromium biosorption and nitrogen removal by the aerobic denitrifying bacterium *Pseudomonas stutzeri* YC-34 from chromium-rich wastewater

**DOI:** 10.3389/fmicb.2022.961815

**Published:** 2022-08-05

**Authors:** Keyin Yang, Huijun Bu, Ying Zhang, Hongxia Yu, Sining Huang, Lixia Ke, Pei Hong

**Affiliations:** College of Life Sciences, School of Ecology and Environment, Collaborative Innovation Center of Recovery and Reconstruction of Degraded Ecosystem in Wanjiang Basin Co-founded by Anhui Province and Ministry of Education, Anhui Normal University, Wuhu, China

**Keywords:** strain YC-34, auto-aggregation, extracellular polymeric substances (EPS), nitrogen removal, Cr(VI) stress

## Abstract

The impact of high concentrations of heavy metals and the loss of functional microorganisms usually affect the nitrogen removal process in wastewater treatment systems. In the study, a unique auto-aggregating aerobic denitrifier (*Pseudomonas stutzeri* strain YC-34) was isolated with potential applications for Cr(VI) biosorption and reduction. The nitrogen removal efficiency and denitrification pathway of the strain were determined by measuring the concentration changes of inorganic nitrogen during the culture of the strain and amplifying key denitrification functional genes. The changes in auto-aggregation index, hydrophobicity index, and extracellular polymeric substances (EPS) characteristic index were used to evaluate the auto-aggregation capacity of the strain. Further studies on the biosorption ability and mechanism of cadmium in the process of denitrification were carried out. The changes in tolerance and adsorption index of cadmium were measured and the micro-characteristic changes on the cell surface were analyzed. The strain exhibited excellent denitrification ability, achieving 90.58% nitrogen removal efficiency with 54 mg/L nitrate-nitrogen as the initial nitrogen source and no accumulation of ammonia and nitrite-nitrogen. Thirty percentage of the initial nitrate-nitrogen was converted to N_2_, and only a small amount of N_2_O was produced. The successful amplification of the denitrification functional genes, *norS, norB, norR*, and *nosZ*, further suggested a complete denitrification pathway from nitrate to nitrogen. Furthermore, the strain showed efficient aggregation capacity, with the auto-aggregation and hydrophobicity indices reaching 78.4 and 75.5%, respectively. A large amount of protein-containing EPS was produced. In addition, the strain effectively removed 48.75, 46.67, 44.53, and 39.84% of Cr(VI) with the initial concentrations of 3, 5, 7, and 10 mg/L, respectively, from the nitrogen-containing synthetic wastewater. It also could reduce Cr(VI) to the less toxic Cr(III). FTIR measurements and characteristic peak deconvolution analysis demonstrated that the strain had a robust hydrogen-bonded structure with strong intermolecular forces under the stress of high Cr(VI) concentrations. The current results confirm that the novel denitrifier can simultaneously remove nitrogen and chromium and has potential applications in advanced wastewater treatment for the removal of multiple pollutants from sewage.

## Introduction

Biological wastewater treatment processes are the most widely used methods for the removal of organic and inorganic pollutants from wastewater treatment technologies (Cai et al., 2020; Nguyen et al., 2021; Uluseker et al., 2021). The advanced nitrate removal is performed by denitrifying functional microbiota, which is the critical process to achieve the standard discharge of nitrogen. The direct addition of functional bacteria to the biological treatment system remains one of the most common methods (Laothamteep et al., 2022; Ma et al., 2022). However, this method yields slow results due to the low initial concentration of functional bacteria compared to native microorganisms, which may result in the loss of functional bacteria (Chen et al., 2015). Auto-aggregation refers to the inter-cellular interaction of bacteria spontaneously gathering to facilitate the attachment of functional microorganisms to the biofilm (Adav et al., 2008; Hong et al., 2020). For biofilm formation, a better option may be to immobilize EPS-producing bacteria on a carrier and then add the bioimmobilized carrier to the reactor (Zhao et al., 2018; Hong et al., 2021). This approach may reduce the loss of functional bacteria and increase the initial concentration of EPS-producing bacteria, thus facilitating biofilm development. Till now, a few aerobic denitrifiers with auto-aggregation ability have been reported, such as, *Klebsiella* sp. TN-10, *Enterobacter* sp. strain FL, and *Methylobacterium gregans* DC-1 (Wei et al., 2016; Fan et al., 2019; Hong et al., 2019). Therefore, the acquisition and application of auto-aggregating strains could have a major impact on accelerating biofilm formation and shortening the start-up time of biofilm reactors.

On the other hand, many municipal wastewater treatment plants are responsible for treating some industrial wastewater and domestic wastewater (Luhar et al., 2021). However, the rapid industrial development has increased the risk of excessive heavy metals discharge from wastewater treatment plants (Wang et al., 2018). The excessive concentration of heavy metal ions may lead to the unstable performance of the wastewater treatment system, lowering the efficiency of the denitrification process (Ochoa-Herrera et al., 2009; Zhou et al., 2021). Among the heavy metals, chromium is one of the most common ones, which is found in wastewater from industries such as steel manufacturing, electroplating, leather tanning, pulp production, landfills, etc. (Truskewycz et al., 2018; Tsybulskaya et al., 2019). Hexavalent chromium easily enters the cytoplasmic matrix through the cell membrane of denitrifying bacteria, which changes the conformation of enzymes and blocks the necessary functional groups of microorganisms, leading to a decrease in the denitrification capacity of bacteria (Colussi et al., 2009; Konovalova et al., 2009). Currently, the inhibitory effect of metal cadmium on denitrification has been alleviated by supplementing bio-promoters such as biotin, cytokinin, and L-cysteine (Wang et al., 2015, 2021; Zhou et al., 2021). However, the addition of exogenous substances may require the creation of new compounds containing the relevant structural units and increase the cost of the denitrification process (Palanivel et al., 2020; Wen et al., 2022). In comparison, heavy metal removal by denitrifying bacteria themselves is a clean, environmentally friendly, and efficient removal strategy (Peng et al., 2019; Hong et al., 2022).

The denitrification process is catalyzed by four enzymes: nitrate reductase (Nar/Nap), nitrite reductase (Nir), nitric oxide reductase (Nor), and nitrous oxide reductase (Nos), encoded by the genes *nar*/*nap, nir, nor* and *nos*, respectively. N_2_O, as an intermediate metabolite, is the third most powerful greenhouse gas after CO_2_ and CH_4_ (Uraguchi et al., 2009). The application of denitrifying bacteria with complete denitrification pathways to reduce N_2_O has become a hot research topic for controlling greenhouse gas emissions from agricultural soils and water bodies (Perez-Garcia et al., 2017; Harris et al., 2021). The release of large amounts of nitrous oxide from denitrifying bacteria would hinder their denitrification applications (Tallec et al., 2008; Miyahara et al., 2010; Shoun et al., 2012). Therefore, such denitrifying bacteria are explored to develop environmentally friendly nitrogen transformation methods.

To reduce functional microbial loss and unstable nitrogen removal under high concentration chromium stress, a novel strain of auto-aggregating denitrifying bacteria, *Pseudomonas stutzeri* YC-34, was screened and obtained in this study. Firstly, the nitrate reduction capability, nitrogen balance, and nitrogen-removal genes of strain YC-34 were analyzed. Secondly, the aggregation property and mechanisms of this strain were revealed by EPS content and spectroscopic measurements, aggregation and hydrophobicity index tests. Thirdly, the tolerance of the strain to Cr(VI) was analyzed, and its potential application was evaluated by experimentally investigating the influence of Cr(VI) on nitrogen removal and EPS production. The research might provide useful information for the development of biotechnological relevant microorganisms to control integrated contamination.

## Materials and methods

### Culture mediums

Enrichment medium (EM, g/L): KNO_3_ 5.0, sodium succinate dibasic hexahydrate11.1, KH_2_PO_4_ 1.0, Na_2_HPO_4_·12H_2_O 7.03, MgSO_4_·7H_2_O 0.13, NH_4_Cl 0.2, trace element solution 2 mL, pH 7.0.

Bromothymol Aroma Blue solid medium (BTB, g/L): KNO_3_ 1.0, trisodium citrate dehydrate 5.3, KH_2_PO_4_ 0.6, FeSO_4_·7H_2_O 0.03, CaCl_2_ 0.1, MgSO_4_·7H_2_O 0.6, 1% bromothymol aroma blue 1 mL, agar 20, pH 7.0.

Nitrogen removal medium (NR, g/L): sodium succinate dibasic hexahydrate11.1, KH_2_PO_4_ 0.1, MgSO_4_·7H_2_O 0.1, KNO_3_ 0.36, trace element solution 2 mL, pH 7.0 (simulation of synthetic wastewater).

Contents of trace element solution (g/L): FeCl_2_·4H_2_O 1.8, CoCl_2_·6H_2_O 0.25, NiCl_2_·6H_2_O 0.01, CuCl_2_·2H_2_O 0.01, MnCl_2_·4H_2_O 0.70, ZnCl_2_ 0.1, H_3_BO_3_ 0.5, Na_2_MoO_4_·2H_2_O, NaSeO_3_·5H_2_O 0.01 (Qing et al., 2018).

### Enrichment cultures and isolation of aerobic denitrifiers

Seed sludge was collected from the Huwanwei wastewater treatment system, located in Hefei, China (117°15'79.81′′E, 31°70′68.10′′N). Five milliliter of the seed sludge was added to a 250 mL triangular flask containing 100 mL of EM and incubated in a shaker at 30°C and 120 r/min for 24 h. Then, 5 mL of culture medium was transferred to a fresh sterile EM medium and the enrichment was repeated for three rounds. The last obtained culture medium was sequentially diluted in a gradient from 10^−1^ to 10^−7^. 0.2 mL of the diluted samples were added to BTB and incubated at 30°C in an incubator until the appearance of single colonies. Single blue colonies were selected, purified by multiple scribing, and stored at 4°C in the refrigerator. Each single purified colony was examined separately using NR, which used nitrate as the only nitrogen source. After comparing the ${\text{NO}}_{3}^{-}-\text{N}$ removal rates, the most efficient colony was labeled YC-34 and cultured in NR for further studies. All media were disinfected at 121°C for 20 m and all tests were performed in three repetitions.

### Determination of denitrification-related indices of the strain

#### Gene amplification

Two milliliter suspension of YC-34 was transferred to 100-mL NR in a 250-mL triangular flask and incubated at 30°C and 120 rpm. After a 24 h culture in NR, a bacterial genomic DNA extraction kit (BK2021081230, DiscoverBeads company, China) was used following the manufacturer's instructions to extract DNA from the strain suspension. The primers and amplification steps for 16S rRNA and denitrification genes are shown in Table 1. PCR products were sequenced by the I-congene Biotechnology company (Wuhan, China) and then analyzed using the BLAST tool of the NCBI database. A phylogenetic tree of the 16S rRNA was constructed by MEGA software (version 6.0). Strain YC-34 has been submitted to the China Center for Type Culture Collection (CCTCC) (Wuhan, China) with the accession number of CCTCC M 20211100).

**Table 1:** The primers and conditions of PCR.

**Genes**	**Primers**	**Conditions of PCR**						
16s rRNA	5'-AGAGTTTGATCCTGGCTCAG-3'/5'-GGTTACCTTGTTACGACTT-3'	95°C, 5 min	94°C, 30 s	69°C, 1 min	72°C, 1 min	35 cycles	72°C, 10 min	Qing et al., 2018
*napA*	5'-TCTGGACCATGGGCTTCAACCA-3'/5'-ACGACGACCGGCCAGCGCAG-3'	95°C, 5 min	94°C, 30 s	69°C, 1 min	72°C, 1 min	35 cycles	72°C, 10 min	Qing et al., 2018
*nirS*	5'-GAACCTCAAGACCACTACCATC-3'/5'-CCTTCCAGTTGTGCTCCTT-3'	95°C, 5 min	94°C, 30 s	57°C, 1 min	72°C, 1 min	35 cycles	72°C, 10 min	Hong et al., 2022
*norR*	5'-GGAAATGACCAAGAACGAGC-3'/5'-AGGTAGCAGACCAGACCGAT-3'	95°C, 5 min	94°C, 30 s	59°C, 1 min	72°C, 1 min	35 cycles	72°C, 10 min	Hong et al., 2022
*nosZ*	5'-GGTAACCTTGACAACACCGA-3'/5'-ATGACGAAGCCGTGAGACA-3'	95°C, 5 min	94°C, 30 s	58°C, 1 min	72°C, 1 min	35 cycles	72°C, 10 min	Hong et al., 2022

#### Nitrogen removal test

Two milliliter culture suspension of strain YC-37 was added to a 250-mL triangular flask containing 100 mL NR and incubated in an incubator shaker (120 rpm) at 30°C. Samples were collected every 6 h to measure the OD_600_ value and the concentrations of TN, ${\text{NH}}_{4}^{+}$, ${\text{NO}}_{2}^{-}$ and ${\text{NO}}_{3}^{-}$. Under sterile conditions, 2 mL of the pre-incubated strain suspension was inoculated into a 250 mL serum bottle containing 100 mL NR. A blank control without inoculation of the bacterial solution was also set up. The serum bottles were aerated with 99.99% pure oxygen, tightly plugged with rubber sealing plugs, and placed in an incubator for 48 h at 30°C. The nitrogen balance was calculated by measuring the starting and final nitrogen content. The starting ${\text{NO}}_{3}^{-}-\text{N}$ content was the initial TN concentration. The final nitrogen measurement indices included TN, ${\text{NH}}_{4}^{+}\text{N}$, ${\text{NO}}_{2}^{-}\text{N}$, ${\text{NO}}_{3}^{-}\text{N}$, organic nitrogen (Org-N), intracellular nitrogen, and gaseous nitrogen concentration. The Org-N concentration was determined by subtracting the ${\text{NO}}_{3}^{-}-\text{N}$, ${\text{NH}}_{4}^{+}-\text{N}$, and ${\text{NO}}_{2}^{-}-\text{N}$ concentrations from the final TN concentration. In addition, the headspace gas sample in the serum bottle was withdrawn and assayed for N_2_O and N_2_ content using GC-MS (Agilent, USA). The bacteria were freeze-dried. The intracellular nitrogen percentage was determined by an elemental analyzer (FLASH 2000, Thermo Fisher Scientific) and the intracellular nitrogen content was calculated by combining the weight of the bacteria.

#### Factors affecting nitrogen removal

NR was used as the tested medium, and the culture conditions were consistent with those described above. The medium composition and condition were adjusted accordingly to the tested variables. Influencing variables included the carbon source, carbon: nitrogen (C/N) ratio (changing the quantity of nitrogen content while maintaining a fixed quantity of carbon content), pH, temperature, and dissolved oxygen (DO, controlled by changing rotation speed). The carbon-based resources (sodium succinate, trisodium citrate, sucrose, sodium acetate, and seignette salt), C/N proportions (5, 10, 15, 30, and 60), pH (5, 7, and 9), temperature (25, 30, and 35°C) and rotational speed (90, 120, and 150 rpm) were chosen as the dependent variables. All testing media were cultured for 30 h, and OD_600_ and ${\text{NO}}_{3}^{-}$ were measured.

### Determination of indicators related to strain aggregation

Samples were taken periodically during the culture process of strain YC-34 in NR to determine indicators related to aggregation. Auto-aggregation and hydrophobicity indices were determined, and EPS extraction was performed with reference to Hong et al. (2019). Briefly, the auto-aggregation index was determined by spectrophotometry after static precipitation, while the hydrophobicity index was measured by spectrophotometry after hexadecane adsorption. Furthermore, EPS was extracted by the cation exchange resin method. The sum of polysaccharides and proteins represented EPS content, which was measured by anthrone colorimetry and the Lowry method separately (Eboigbodin and Biggs, 2008). EPS was treated with freezing intervention and then ground with infrared grade KBr powder, made into disks. Subsequently, Fourier transform infrared spectroscopy (FTIR) was used for measurement (Nicolet Nexus, Thermo, USA).

### Determination of cadmium biosorption-related indicators

Bulk tests were conducted in an aseptic NR medium to investigate the ability of strain YC-34 to remove ${\text{NO}}_{3}^{-}$ in the presence of Cr(VI). According to reports, wastewater with Cr(VI) usually contained <10 mg/L (Das et al., 2016; Sharma and Malaviya, 2016). Therefore, the initial cadmium concentration in the culture medium was adjusted to 0, 3, 5, 7, and 10 mg/L by adding the corresponding concentrations of potassium dichromate to the NR medium. Pre-cultured YC-34 was incubated (1%, v/v) in an NR medium containing different concentrations of Cr(VI) at 30°C and 120 rpm. After 48 h of culture, the content and composition of EPS, OD_600_, and TN were measured, and the nitrate and Cr(VI) content were determined. The 1,5-Diphenylcarbazide spectrophotometric method was used to mearsure the concentration of Cr(VI) (He et al., 2015) while the total Cr concentration was determined by atomic absorption spectrometry (AA-7003, EWAI, Beijing, China). The Cr(III) was evaluated by subtracting Cr(VI) from the total Cr (An et al., 2020). After fixing in aqueous 2.5% glutaraldehyde for 12 h and gradient dehydration with different concentrations of ethanol, the cells were observed under a scanning electron microscope (SEM, Hitachi, Japan) (Hong et al., 2019).

### Analytical methods

The concentrations of TN, ${\text{NH}}_{4}^{+}-\text{N}$, ${\text{NO}}_{3}^{+}-\text{N}$, and ${\text{NO}}_{2}^{-}-\text{N}$ were measured with reference methods described in the Chinese national standards (NY525-2012). The amide I region (1,700–1,600 cm^−1^) of the FTIR data was analyzed to extract information regarding protein secondary structures (Jia et al., 2017). In addition, secondary derivative spectroscopy and deconvolution spectroscopy of the amide I region and type of hydrogen bonding in the region of 3,000–3,800 cm^−1^ were performed using Peakfit software (version 4.12). SPSS 19.0 software (IBM SPSS, Armonk, NY, USA) was used for all data processing and statistical analyses. Line and bar charts were drawn using Origin 2021 (Origin Lab, Northampton, MA, USA).

## Results and discussion

### Identification and characterization of YC-34

**Figure 1:**
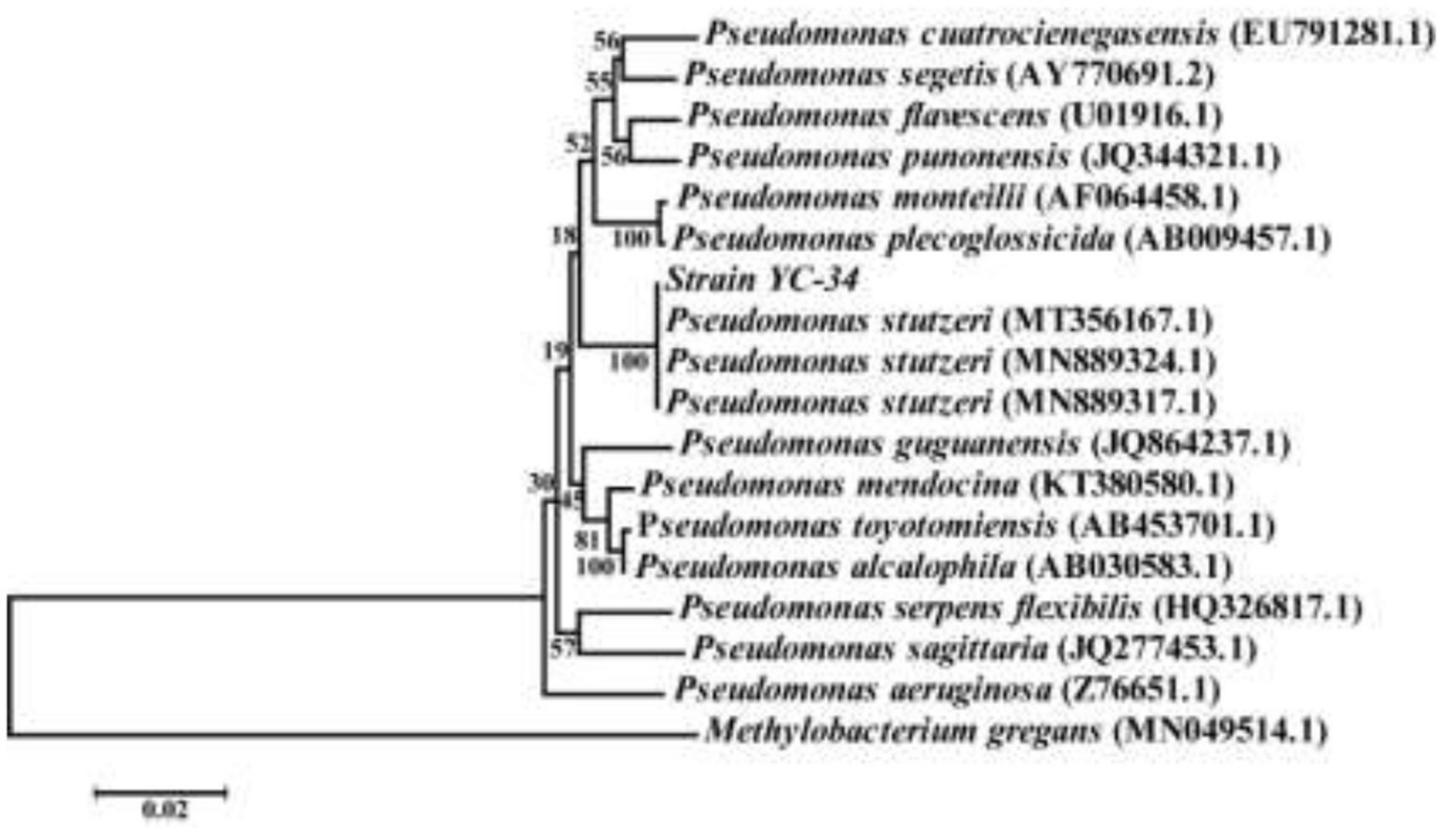
The phylogenetic tree of *Pseudomonas stutzeri* strain YC-34 and related strains.

After multiple cycles of enrichment in EM and plate scribing on BTB solid medium, the auto-aggregation denitrifier YC-34 was obtained. The strain was off-white, convex, and opaque, with a smooth, moist, and thick surface on BTB. PCR amplification results revealed that the whole length of the 16S rRNA sequence of YC-34 was ~1,375 bp (GenBank number: MZ855228). YC-34 was found to be highly associated with *Pseudomonas sp*. strain SM12 (GenBank number: MT356167), with 99% similarity. Phylogenetic analysis based on 16S rRNA gene sequencing indicated that YC-34 had a close relationship with *Pseudomonas stutzeri* (Figure 1). Therefore, strain YC-34 was identified as a *Pseudomonas stutzeri* strain.

### Analysis of nitrogen-removal characteristics

**Figure 2:**
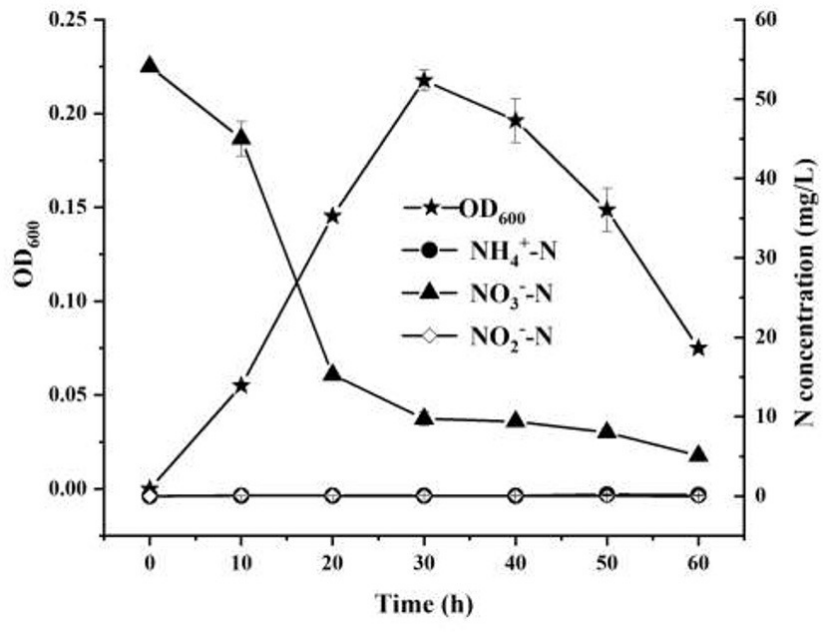
The nitrate removal and cell growth performance of YC-34 strain.

The nitrogen removal characteristics of strain YC-34 were analyzed by using nitrate as the single nitrogen source (Figure 2). From 0 to 60 h, ${\text{NO}}_{3}^{-}-\text{N}$ was reduced from the original 54.12–5.10 mg/L with an elimination efficiency of 90.58%. Moreover, the accruals of nitrite and ammonia were almost zero during the whole incubation period. *Pseudomonas stutzeri* strains were previously reported as aerobic denitrifying bacteria with the ability to accumulate nitrite (Zhu et al., 2012; Hong et al., 2021). However, nitrite enrichment inhibits the functions of microorganisms in the nitrogen, phosphorus, and sulfate removal process, such as anaerobic ammonium oxidation bacteria, methanogenic archaea, and sulfate-reducing bacteria (Auguet et al., 2016; Wang et al., 2019). Therefore, due to its efficient nitrate-nitrogen removal and minimal accumulation of ammonia-nitrogen and nitrite, strain YC-34 showed excellent denitrification performance.

**Figure 3:**
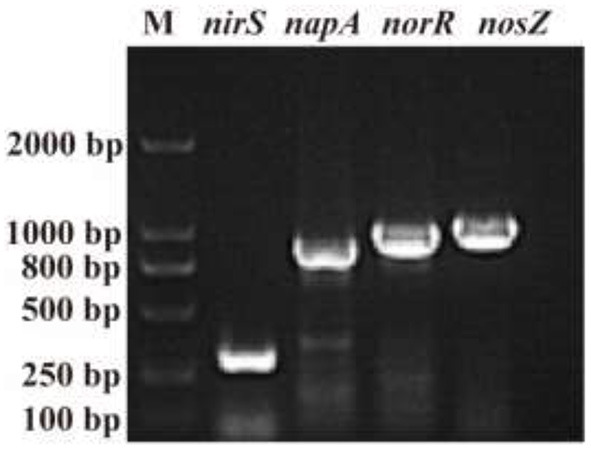
The amplification results of *nirS, norR, nosZ*, and *napA* genes (M: DL 2000 DNA marker).

The N conversion pathway for strain YC-34 was explored by N balance. The N balance data are shown in Table 2. Comparing the initial and final nitrogen concentrations, 31.1% of the original nitrate was transformed into intracellular nitrogen, 17.8% was converted into organic nitrogen, 8.5% was turned into N_2_O, and 30.0% was transformed into N_2_. As described by Huang et al. (2015), the denitrification procedure requires the participation of multiple enzymes. The related genes n*apA, nirS, norR* and *nosZ* were amplified and were found to be 877, 310, 1,001, and 1,051 bp, respectively (Figure 3). The genes corresponded to four enzymes (NAP, NIR, NOR, and NOS). NAP played an essential role in the conversion of ${\text{NO}}_{3}^{-}$ to ${\text{NO}}_{2}^{-}$ (Zhu et al., 2012). The *napA* gene is often used as a functional marker to identify aerobic denitrifying bacteria (Feng et al., 2018; Lang et al., 2019; Zhang et al., 2019). The amplification of the *nirS* gene indicates that heme c in strain YC-34 is responsible for electron transport from the electron donor cytochrome c551, while heme d1 is responsible for nitrite binding and reduction to nitric oxide (Baker et al., 1997). The enzymes NOR and NOS are encoded by the *norR* and *nosZ* genes, respectively, which promote the production of N_2_O and N_2_, respectively (Zhang et al., 2012). The nitrate-nitrogen removal pathway of strain YC-34 was like the reported strains, *Pseudomonas stutzeri* strain XL-2; *Pseudomonas stutzeri* KY-37; *Pseudomonas oligotrophica* JM10B5a^T^ (Zhao et al., 2018; Hong et al., 2022; Zhang et al., 2022). Combined with the nitrogen balance and denitrification gene amplification, strain YC-34 exhibited a complete N pathway: Nitrate → Nitrite → Nitric coxide → Nitrous oxide → Nitrogen.

**Table 2:** The N balance of strain YC-34 after 48 h cultivation.

**Initial N (mg)**	**Final N (mg)**							**Error**
	** ${\text{NO}}_{3}^{-}-\text{N}$ **	** ${\text{NO}}_{2}^{-}-\text{N}$ **	** ${\text{NH}}_{4}^{+}-\text{N}$ **	**Org-N**	**Intracellular N**	**N_2_O**	**N_2_**	
2.17 ± 0.008	0.07 ± 0.005	UN	0.003	0.48 ± 0.009	0.84 ± 0.011	0.28 ± 0.015	0.81 ± 0.005	7.8%

UD, Undetectable.

Error% = 100× (Initial N-Final N)/Initial N.

### Effects of different influencing factors on the denitrification performance of YC-34

#### Carbon source

**Figure 4:**
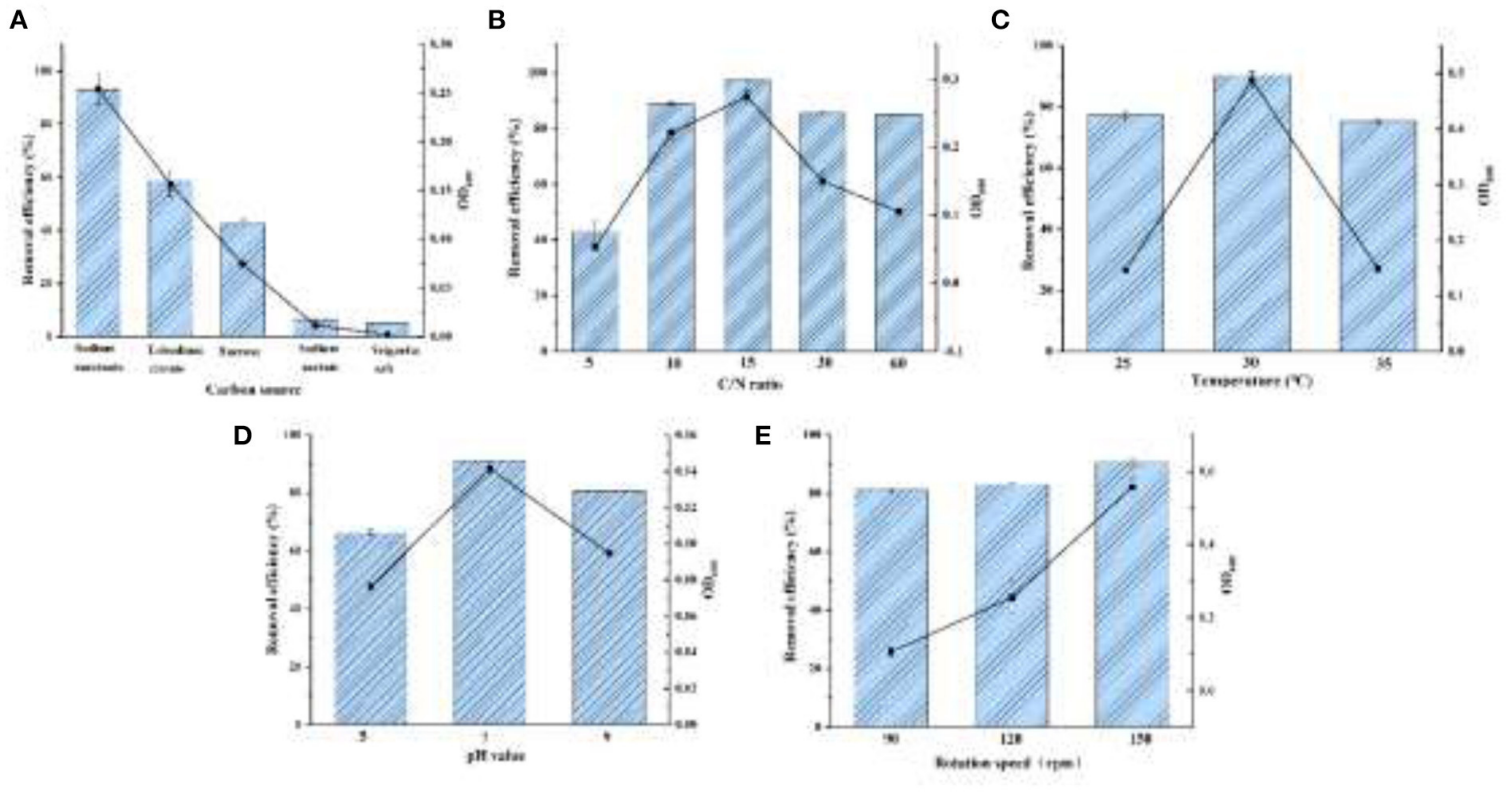
The effects for denitrification capacity by the conditions of carbon source **(A)**, C/N ratio **(B)**, temperature **(C)**, pH **(D)**, and DO **(E)**.

As shown in Figure 4A, sodium succinate, trisodium citrate, and sucrose were tested for strain YC-34 growth with a ${\text{NO}}_{3}^{-}$ elimination efficiency of 93.08, 58.72, and 42.51%, respectively. Sodium succinate might be the optimal carbon source, which consisted of the carbon using of *Bacillus methylotrophicus* strain L7 (Zhang et al., 2012).

#### C/N ratio

Aerobic denitrifiers usually require a C/N ratio of about 9–10 (Ren et al., 2021). The removal of nitrate by *Halomonas Alkaliphile* HRL-9 with a C/N ratio of 20 was significantly higher than that of 10 (Ren et al., 2019). As displayed in Figure 4B, YC-34 could adapt to C/N ratios ranging from 5 to 60. As the ratio gradually increased from 5 to 60, the utilization of ${\text{NO}}_{3}^{-}$ initially presented an increasing trend followed by a decreasing trend. YC-34 reached its peak efficiency (97.37% ${\text{NO}}_{3}^{-}$ removal) at a C/N ratio of 15.

#### Temperature

YC-34 could maintain efficient nitrogen removal from 25 to 35°C (Figure 4C). The maximum removal capacity of ${\text{NO}}_{3}^{-}$ reached 90.43% at 30°C, which was similar to the *Marinobacter* strain NNA5 (Liu et al., 2016). Moreover, the OD_600_ of the strain is higher than that of other temperatures under the condition of 30°C. This indicated that YC-34 might be a mesophilic strain.

#### pH

Figure 4D presented the ${\text{NO}}_{3}^{-}$ removal properties of YC-34 under the initial pH of 5, 7, and 9 with a maximum removal value of 66.30, 91.10, and 80.82%, respectively. The optimal pH condition of YC-34 was similar to that of *Acinetobacter* sp. YT03, which maintained a high nitrogen removal capacity at a pH of 7 (Li et al., 2019). This indicated that the optimum pH for YC-34 growth was neutral.

#### Shaking speed

An increase in shaking speed represents an increase in DO. The denitrification rate of strain *Acinetobacter* sp. YT03 was reported to increase as the rotation speed increased. As the rotation speed was increased from 50 rpm to 250 rpm, the nitrogen removal rate reached the maximum value of 93.9% at 250 rpm (Li et al., 2019). The strain YC-34 showed a similar performance to *Acinetobacter* sp. YT03, which showed a maximum nitrogen removal efficiency of 90.4% at 150 rpm (Figure 4E). According to the experimental results, sodium succinate was the most suitable carbon source for YC-34. The optimum C/N ratio was 15, the suitable temperature was 30°C, and the optimum pH was 7. YC-34 also showed outstanding denitrification performance in suboptimal factors, which confirmed the strong environmental adaptability of YC-34.

### Analysis of prominent auto-aggregation features and mechanisms

#### Auto-aggregation ability and hydrophobicity of YC-34

**Figure 5:**
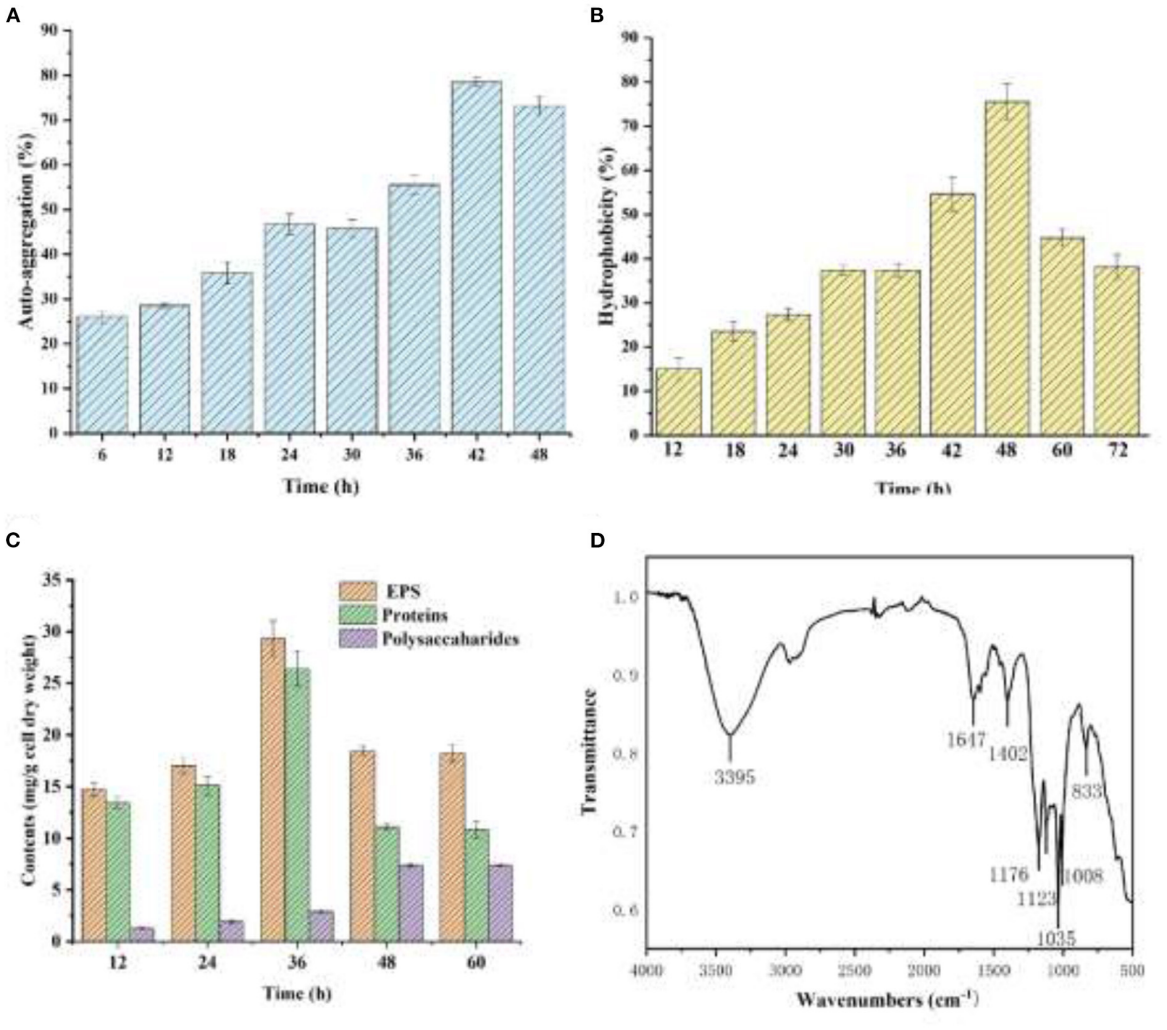
Auto-aggregation index **(A)** and hydrophobicity index **(B)** of the strain YC-34, Fourier transform infrared spectra **(C)** and composition **(D)** of EPS.

As shown in Figure 5A, YC-34 presented a significant auto-aggregation ability as the auto-aggregation index progressively rose to 78.4% at 42h. The value was higher than that of the *Enterobacter* sp. strain FL and *Escherichia coli* MG1655 (Eboigbodin and Biggs, 2008; Wang et al., 2018). The surface hydrophobicity of YC-34 gradually increased from 15.0% at 6 h to 75.5% at 48 h (Figure 5B), which was significantly higher than that of *Sphingomonas* sp. YY2 (Lang et al., 2019). These results were consistent with *Bifidobacteria* with its hydrophobicity presenting a positive correlation with aggregation ability (Collado et al., 2007).

#### EPS characteristics

As presented in Figure 5C, the protein and polysaccharide concentrations rose progressively from 12 to 36 h. YCh.... t-Yc-34 was rich in proteins. Figure 5D displays the infrared wavebands of EPS. The 3,395 cm^−1^ peaks represented the tensile oscillation of O-H. The peak of 1,647 cm^−1^ corresponded to the C=O stretching oscillation of amide-I, which was identified as the random coil of protein secondary structure. The peak at 1,402 cm^−1^ was attributed to the COO-deformation vibration due to the presence of uronic acid. The wavebands at 800 and 1,200 cm^−1^ were attributed to the C-H deformation vibration. The results confirmed that the main components of EPS produced by YC-34 were proteins and polysaccharides. To investigated the effect of proteins in the auto-aggregation of YC-34, the primary structure of proteins was further studied. As shown in Table 3, the secondary structures were composed of 37.07% β-sheets and 62.93% β-turns, whereas the α-helix structures and random coil were not found. There was a higher content of β-turn than β-sheet. Fewer α-helices in the protein resulted in a “loose” protein structure, exposing more hydrophobic amino acids, leading to stronger hydrophobicity. The auto-aggregation ability and surface hydrophobicity of bacteria were closely related to biofilm formation (Wang and Li, 2022). Proteins and polysaccharides in EPS were essential in promoting initial bacterial adhesion and biofilm development (Zhu et al., 2018). Replenishment of EPS-producing bacteria in wastewater biofilm treatment systems may facilitate EPS production, and enhance initial adhesion and biofilm development, and eventually leading to accelerated biofilm formation. This would reduce the loss of nitrogen removal functional bacteria, and serve to achieve enhanced deep nitrogen removal from wastewater.

**Table 3:** The content of protein secondary structure of strain YC-34.

	**β-Sheets (%)**	**Random coil (%)**	**α-Helices (%)**	**β-turn (%)**
EPS	37.07%	—	—	62.93%

### Analysis of the effect and mechanism of Cr (VI)-removal by YC-34

**Figure 6:**
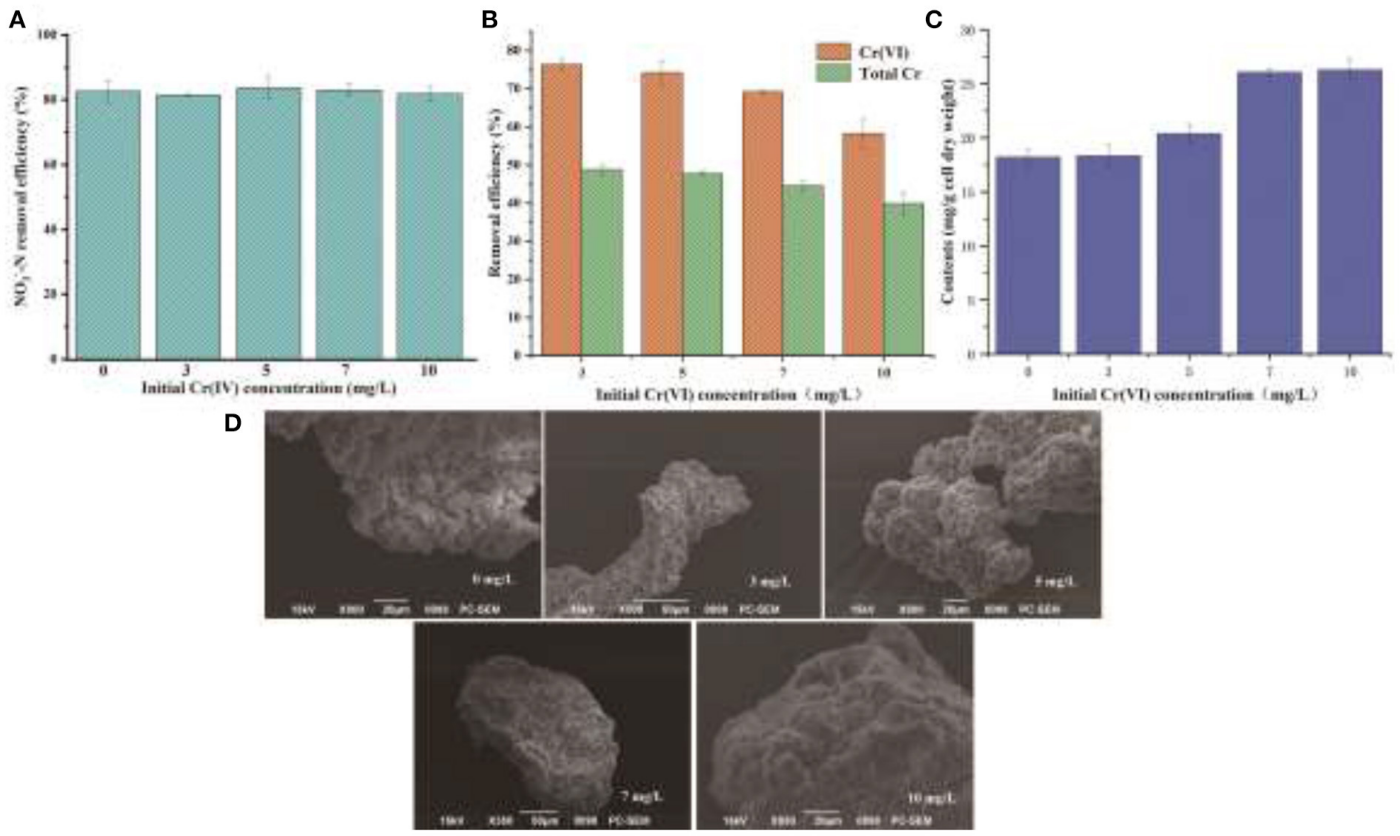
Denitrification capacity **(A)**, Cr removal **(B)**, EPS generation **(C)** under the different concentrations of Cr(VI); morphology of EPS at different Cr(VI) concentrations of 0, 3, 5, 7, and 10 mg/L under SEM observation **(D)**.

Some previous studies have shown that EPS has various binding and biosorption capacities for different kinds of heavy metals (Yue et al., 2015). In view of a large amount of EPS secretion by YC-34, the nitrogen removal characteristics of YC-34 in response to heavy metal Cr(VI) stress were further investigated. As presented in Figure 6A, the ${\text{NO}}_{3}^{-}-\text{N}$ removal efficiency of YC-34 was 82.6, 81.5, 83.6, 83.0, and 81.8% at initial Cr(VI) concentrations of 0, 3, 5, 7, and 10 mg/L, respectively. After 48 h of incubation, the total Cr concentrations decreased to 1.54, 2.62, 3.88, and 6.02 mg/L, respectively (Figure 6B). Chromium loss might result from the biosorption of YC-34, with the adsorption efficiency reaching 48.75, 46.67, 44.53, and 39.84% at 3, 5, 7, and 10 mg/L initial Cr(VI) contents, respectively. By comparing the reduction traits of Cr(VI) and total Cr, strain YC-34 showed similar chromium removal characteristics to strain AL-6, converting hexavalent chromium to the less toxic Cr(III) (An et al., 2020).

**Figure 7:**
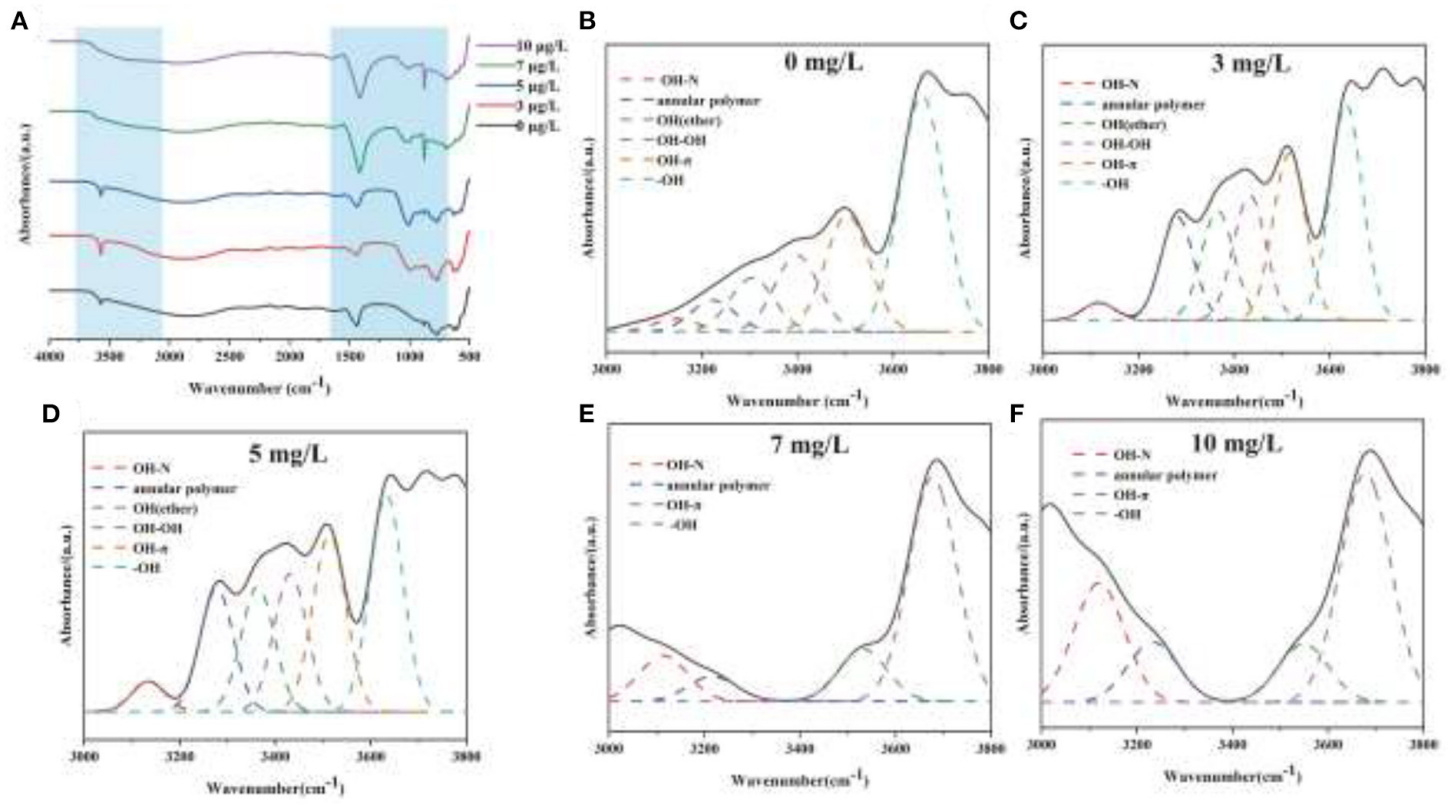
FT-IR spectra of strain YC-34 under different Cr(VI) stress **(A)** and their deconvoluted results for 0 **(B)**, 3 **(C)**, 5 **(D)**, 7 **(E)**, and 10 mg/L **(F)** at the region (3,000–4,000 cm^−1^).

**Figure 8:**
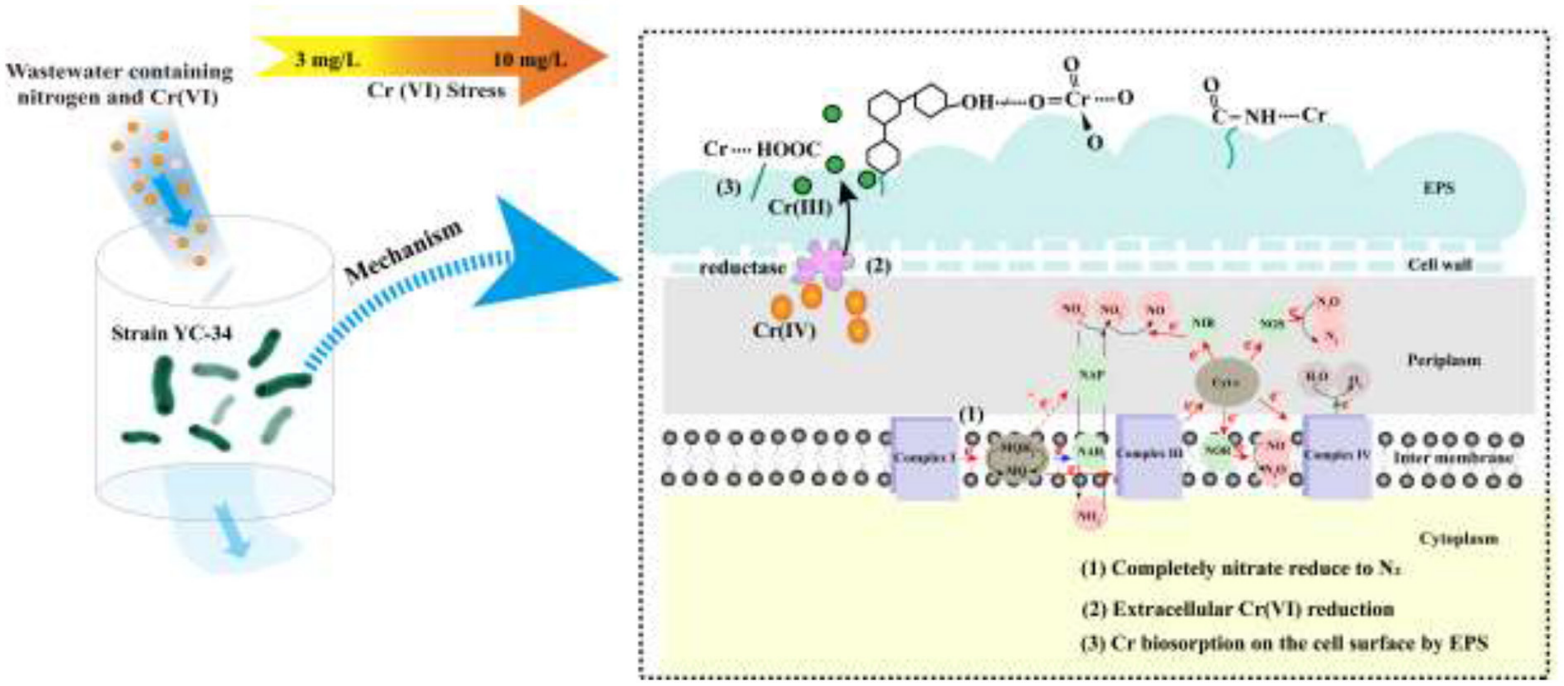
The speculative nitrate conversion and Cr(VI) removal mechanism of strain YC-34.

The mechanism of chromium adsorption by strain YC-34 was further explored from the micro-characteristics of the cell surface. The accumulation of EPS increased with increasing Cr(VI) concentration, reaching a maximum of 26.32 mg/g cell dry weight under the initial 10 mg/L Cr(VI) condition (Figure 6C). The EPS encapsulation of Cr(VI) by strain YC-34 was observed under SEM (Figure 6D), which was similar to the results of Zhou et al. (2021). The adsorptive removal of chromium by strain YC-34 may be achieved through adsorption sites on EPS (Jin et al., 2014; Pi et al., 2020), while the large amount of EPS production helps strains to establish a stable structure that protects them from hazardous environments (Miao et al., 2018). Moreover, FTIR measurements and analysis showed almost no change in the transmittance of the measured bands of strianYC-34 in the groups with initial Cr(IV) of 0, 3 and 5 mg/L (Figure 7). Cell surface polymers are generally supported by a hydrogen bonding system, and the higher the proportion of hydrogen bonds, the stronger the intermolecular interactions (Cai et al., 2021). Further deconvolution analysis of FT-IR results in the 3,800–3,000 cm^−1^ band showed that the hydrogen bonding types were significantly higher in the 0, 3, and 5 mg/L groups than in the 7 and 10 mg/L groups, implying that the intermolecular forces on the bacterial surface weakened at Cr(IV) concentrations above 5 mg/L. In summary, considering the nitrogen removal pathway and the extracellular Cr(IV) adsorption characteristics analysis (Figure 8), the main removal mechanisms of strain YC-34 facing cadmium stress may be due to the adsorption of functional groups on the surface.

## Conclusion

A novel auto-aggregation aerobic denitrifier (*Pseudomonas stutzeri* strain YC-34) was isolated, demonstrating superior environmental adaptability and the ability to remove Cr(VI) in synthetic wastewater. YC-34 attained a high ${\text{NO}}_{3}^{-}-\text{N}$ removal efficiency of 90.58% and showed fine adaptability to different culture conditions. Based on nitrogen balance and denitrification gene amplification analysis, the strain YC-34 presented a complete nitrogen pathway for Nitrate → Nitrite → Nitric oxide → Nitrous oxide → Nitrogen. Strain YC-34 produced a large amount of EPS, especially when exposed to Cr(VI), which in turn provided more abundant functional groups and strong hydrogen bonds to adsorb cadmium. These studies indicated that YC-34 has a superior potential for simultaneously treating synthetic wastewater contaminated with nitrogen and Cr(VI).

## Data availability statement

The datasets presented in this study can be found in online repositories. The names of the repository/repositories and accession number(s) can be found in the article/supplementary material.

## Author contributions

PH: conceptualization, methodology, software, data curation, writing-original draft, and writing-review and editing. KY: writing-original draft, methodology, and data curation. HB, YZ, HY, and SH: data curation and investigation. LK: conceptualization, methodology, supervision, and writing-review and editing. All authors contributed to the article and approved the submitted version.

## Funding

This research was supported by Key Research and Development Projects in Anhui Province (202104i07020001), National Natural Science Foundation of China (42107079), Anhui Natural Science Foundation (2108085QD159), the University Synergy Innovation Program of Anhui Province (GXXT-2020-075), Natural Science Foundation of Universities of Anhui Province (KJ2020A0076), and Anhui Normal University Student Innovation and Entrepreneurship Training Program.

## Conflict of interest

The authors declare that the research was conducted in the absence of any commercial or financial relationships that could be construed as a potential conflict of interest.

## Publisher's note

All claims expressed in this article are solely those of the authors and do not necessarily represent those of their affiliated organizations, or those of the publisher, the editors and the reviewers. Any product that may be evaluated in this article, or claim that may be made by its manufacturer, is not guaranteed or endorsed by the publisher.
